# Improved Electrical Characteristics of Gallium Oxide/P-Epi Silicon Carbide Static Induction Transistors with UV/Ozone Treatment Fabricated by RF Sputter

**DOI:** 10.3390/ma14051296

**Published:** 2021-03-08

**Authors:** Myeong-Cheol Shin, Young-Jae Lee, Dong-Hyeon Kim, Seung-Woo Jung, Michael A. Schweitz, Weon Ho Shin, Jong-Min Oh, Chulhwan Park, Sang-Mo Koo

**Affiliations:** 1 Department of Electronic Materials Engineering, Kwangwoon University, 20 Kwangwoon-ro, Nowon-gu, Seoul 01897, Korea; smc0753@naver.com (M.-C.S.); yjmjcj@naver.com (Y.-J.L.); gogomatt@kw.ac.kr (D.-H.K.); swjung0819@naver.com (S.-W.J.); michael.schweitz@schweitzlee.com (M.A.S.); weonho@kw.ac.kr (W.H.S.); jmOH@kw.ac.kr (J.-M.O.); chpark@kw.ac.kr (C.P.); 2 Department of Electronic Materials Engineering, Nagoya Institute of Technology, Gokiso-cho, Showa-ku, Nagoya, Aichi 466-8555, Japan

**Keywords:** gallium oxide, silicon carbide, static induction transistor, UV/ozone treatment

## Abstract

In this study, static induction transistors (SITs) with beta gallium oxide (β-Ga_2_O_3_) channels are grown on a p-epi silicon carbide (SiC) layer via radio frequency sputtering. The Ga_2_O_3_ films are subjected to UV/ozone treatment, which results in reduced oxygen vacancies in the X-ray photoelectron spectroscopy data, lower surface roughness (3.51 nm) and resistivity (319 Ω·cm), and higher mobility (4.01 cm^2^V^−1^s^−1^). The gate leakage current is as low as 1.0 × 10^−11^ A at V_GS_ = 10 V by the depletion layer formed between n-Ga_2_O_3_ and p-epi SiC at the gate region with a PN heterojunction. The UV/O_3_-treated SITs exhibit higher (approximately 1.64 × 10^2^ times) drain current (V_DS_ = 12 V) and on/off ratio (4.32 × 10^5^) than non-treated control devices.

## 1. Introduction

Among the different polytypes of Ga_2_O_3_ (α, β, γ, δ, and ε), monoclinic Ga_2_O_3_ is the most thermally stable and can be obtained by annealing sputtered films [1,2,3,4] because metastable Ga_2_O_3_ can be subsequently converted into β-Ga_2_O_3_ after high-temperature annealing. Gallium oxide (Ga_2_O_3_) is a wide-bandgap material with an energy gap (Eg) of 4.8–5.1 eV and has drawn considerable attention for use in various applications such as high-powered semiconductor devices, ultra-violet (UV) detectors, and gas and fire sensors [5,6,7,8,9,10]. In addition, it exhibits an n-type conductivity that does not require intentional doping due to inherent oxygen vacancies in the grown crystal, as in the case of ZnO or InGaZnO (IGZO) [11,12]. To improve rectification or bipolar device elements in Ga_2_O_3_, an n-type Ga_2_O_3_ film should be grown on a p-type substrate.

4-H silicon carbide (SiC) is a wide-bandgap material (Eg ~ 3.26 eV) that clearly shows a higher value of thermal conductivity (~370 W/m·K) than those of Ga_2_O_3_ (~27 W/m·K) and GaN (~177 W/m·K). The maturity of the bulk SiC substrate technology and optimized device processing makes it one of the most desirable candidates for a p-type doped counterpart to form heterojunctions with Ga_2_O_3_ and other materials [13,14,15,16].

Some studies have reported the electron mobility and crystalline quality of Ga_2_O_3_ grown on GaN substrates [17]. The static induction transistor (SIT) is a short-channel field effect transistor that typically displays unsaturated output characteristics [18] and shows potential for use in high frequency and high-powered applications. Consequently, SIT devices could be used for driver circuits such as in switching mode power supplies (SMPS) [19]. Furthermore, Ga_2_O_3_ SIT structures have been formed by using GaN, which exhibits a significant level of gate leakage current [20,21]. This may be controlled by employing a reliable PN heterojunction gate element.

The lattice constants of β-Ga_2_O_3_, *a* = 12.23 Å, *b* = 3.04 Å, *c* = 5.80 Å, and β = 103.7°, result in a lattice mismatch of ~2.63% between the c-plane GaN and β-Ga_2_O_3_ [22]. Note that the lattice mismatch between β-Ga_2_O_3_ grown on a 4H-SiC wafer (*a* = 3.10 Å and *c* = 10.53 Å) can become as low as 3.22%. 4H-SiC can be selected as a growth substrate not only because it has a low lattice mismatch with Ga_2_O_3_ but also because of its high inertness and high thermal conductivity [18].

So far, there have been only limited reports on Ga_2_O_3_/SiC structures, which mostly focus on simple diode structures [23]. Thus, it is important to realize reliable heterojunction device structures of Ga_2_O_3_ and other p-type doped materials.

In this work, we demonstrate SITs fabricated by depositing n-type Ga_2_O_3_ on an epitaxially grown layer of p-type 4H-SiC on an n-type 4H-SiC substrate. The PN heterojunction gate of the SIT forms a depletion layer with a proper gate bias, thereby controlling the Ga_2_O_3_ channel. Our specific goal was to examine the control of gate leakage current through a PN heterojunction gate and the influence of UV/ozone (UV/O_3_) treatment on the performance of SITs.

## 2. Materials and Methods

**Figure 1:**
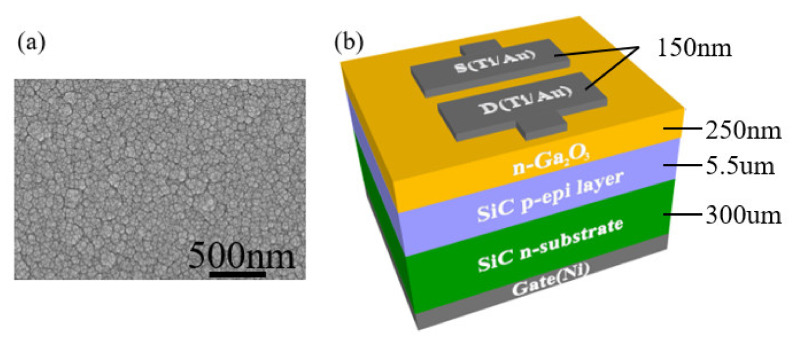
Gallium oxide (Ga_2_O_3_)/4H-silicon carbide (SiC) static induction transistor (SIT) device structure and SEM image. (**a**) SEM data of Ga_2_O_3_, (**b**) SIT device structure.

We used a 4H-SiC wafer with a layer of epitaxially grown 4H-SiC (p-type) on highly doped n-type 4H-SiC. After radio corporation of america (RCA) cleaning the SiC wafer with SPM solution (H_2_SO_4_:H_2_O_2_ = 4:1), we removed the native silicon dioxide (SiO_2_) layer using a buffered oxide etch (BOE) solution. The 150 nm gate nickel film was deposited by E-beam evaporator. The samples were annealed at 1050 °C in N_2_ gas for 1 min by rapid thermal annealing (RTA) to form an ohmic contact with nickel silicide. Ga_2_O_3_ films were deposited by radio frequency (RF) sputtering of a Ga_2_O_3_ (99.99% purity) target. Films of 250 nm were grown on the epitaxial 4H-SiC layer under 35 mTorr at a pure argon mass flow rate of 4 sccm. After deposition, SiC wafers with Ga_2_O_3_ film were annealed at 800 °C for 40 min under N_2_. The Ga_2_O_3_ thin film doping concentration is ~3.27 × 10^15^ cm^−3^, with a resistivity of ~7.39 Ω·cm as measured by hall measurement as confirmed. The Ga_2_O_3_ films were illuminated by UV/O_3_ exposure for comparison with samples, depending on the treatment. Shown in Figure 1, Ti (50 nm) and Au (100 nm) metal was deposited on the Ga_2_O_3_ layer under 6 × 10^−2^ mTorr by E-beam evaporation. The gold layer was applied to prevent the oxidation of the titanium. After Ti/Au-deposition, the samples were annealed at 300 °C for 30 min to from ohmic contacts. 

The SIT consisting of deposited Ga_2_O_3_ on 4H-SiC (doping concentration ~5 × 10^16^cm^−3^) was investigated and the manufactured devices were exposed to UV/O_3_ for varying durations to improve the Ga_2_O_3_ film quality. We used an AHTECH AH-1700 UV ozone cleaner to remove contamination with a wavelength 184 nm and 254 nm. The material properties of Ga_2_O_3_ were characterized by atomic force microscopy (AFM), X-ray photoelectron spectroscopy (XPS), and Hall measurements with UV/O_3_ treatment for different times. I_D_-V_D_ and I_D/G_-V_G_ measurements were obtained and analyzed. The gate voltage-dependent operation of static induction devices will be discussed in the following section.

## 3. Results and Discussion

**Figure 2:**
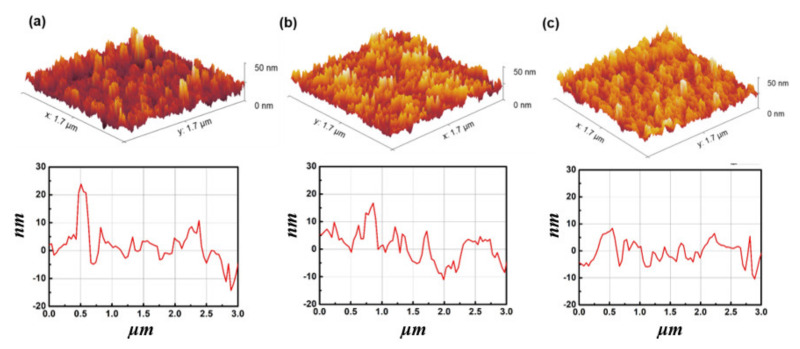
Atomic force microscopy (AFM) images of Ga_2_O_3_ films on 4H-SiC wafers at different times of UV/ozone (UV/O_3)_ exposure: (**a**) non-treated, (**b**) 15 min, and (**c**) 30 min.

Figure 2a–c shows that the surface roughness of the films became smoother as the exposure time increased from 0 to 15 to 30 min; the root-mean-square (RMS) values were 5.23, 4.24, and 3.51 nm, respectively. The light energies at wavelengths of 185 nm and 254 nm energy were larger than contamination bonding energies. Therefore contaminants or organic materials could be removed through UV/O_3_ treatment [24]. If the UV/O_3_ treatment is applied for more than 30 min, the further benefits of the treatment are small [24]. This observation suggests that UV/O_3_ treatment can remove the contamination and organic residue resulting from oxygen radicals [25,26].

**Figure 3:**
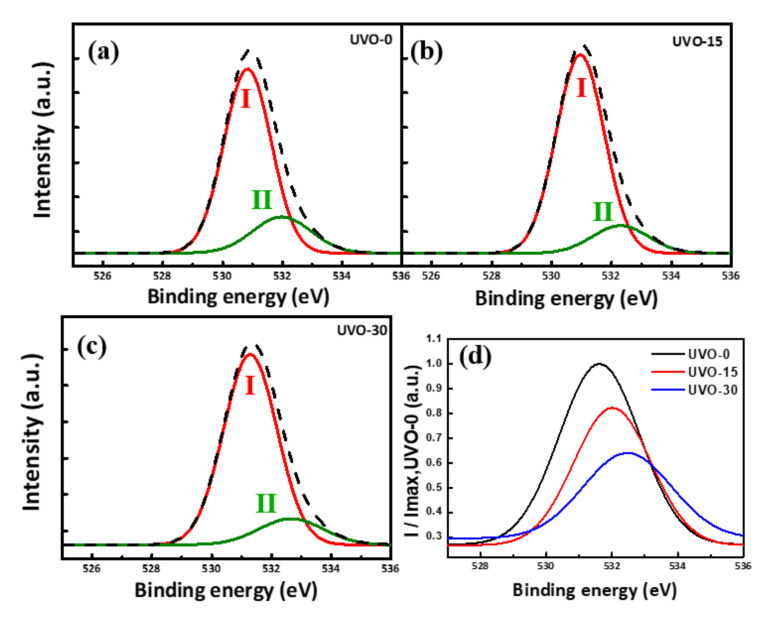
Normalized O 1s X-ray photoelectron spectroscopy (XPS) profiles of Ga_2_O_3_ films: (**a**) non-treated, (**b**) 15 min, and (**c**) 30 min treated. (**d**) Comparison of the extracted O_II_ spectra after UVO-0, UVO-15 and UVO-30 treatments with UV-light.

The bulk and surface defects, which can be observed as surface roughness and oxygen vacancies, may affect the performance of oxide semiconductor devices. During the UV/O_3_ treatment, long-wavelength UV radiation was absorbed by the ozone and decomposed this to form highly reactive atomic oxygen. The atomic oxygen in turn reacted with oxygen vacancies in the thin film, thereby reducing the number of charge carrier traps. The O 1s core-level X-ray photoelectron spectroscopy (XPS) profiles shown in Figure 3 exhibit peaks centered at 528.9 eV (O_I_), which indicate that O_2_ ions bonded with Ga atoms, forming Ga_2_O_3_. It is desirable to maximize metal–oxygen–metal (M-O-M) bonding in an oxide semiconducting layer, which may result in decreased oxygen vacancies. The comparison of the XPS O1s subpeaks is therefore important to understand the behavior. The high M-O-M peaks in the XPS data show that the carrier transport property of the film may have been improved as to the carrier transport properties. The decrease in the oxygen vacancies may have resulted in easier electron transition from the valance band to the conduction band due to the special electron configuration of 3d^10^4p^1^ [27]. The peak with a binding energy of 530.3 eV (O_II_) is associated with the oxygen-deficient regions on the surface of the Ga_2_O_3_ films, which represent the quantity of related oxygen vacancies. Whereas it is possible that further electron states may have made small contributions to the observed O 1s spectra in Figure 3a–c, the extracted O_II_ peaks in (d) describe the relative contribution of the O_II_ states. As shown in Figure 3d the oxygen vacancy peak (O_II_) was reduced by UV/O_3_ treatment. The magnitudes of the O_II_ peaks of the devices treated with UV/O_3_ for 15 min and 30 min were at 80% and 60% of the O_II_ peak magnitude of the untreated sample device, respectively. The decreased oxygen vacancies led to a high Ion/Ioff ratio, mobility, and unintentional doping concentrations.

**Figure 4:**
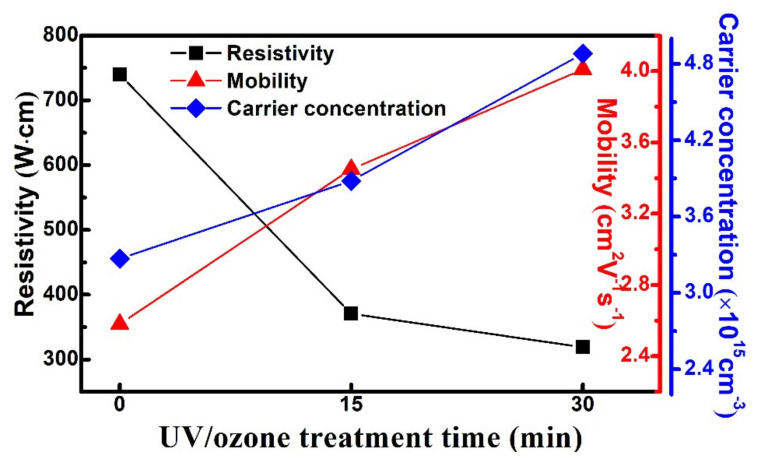
Resistivity, hall mobility, and carrier concentration of Ga_2_O_3_ films deposited on 4H-SiC at different UV/O_3_ treatment times.

Hall effect measurements were performed at room temperature to determine the electrical parameters of the Ga_2_O_3_ films on 4H-SiC. Figure 4 shows the Hall mobility, carrier concentration, and resistivity of the deposited Ga_2_O_3_ films on 4H-SiC as a function of the UV/O_3_ treatment time. The resistivity of Ga_2_O_3_ films decreased from 740 to 319 Ω·cm as the UV/O_3_ treatment time increased. The mobility and n-type carrier concentration increased from 2.58 cm^2^V^−1^s^−1^ and 3.27 × 10^15^ cm^−3^ to 4.01 cm^2^V^−1^s^−1^ and 4.88 × 10^15^ cm^−3^, respectively. The improvement in mobility and carrier concentration was associated with decreasing oxygen-related traps on the Ga_2_O_3_ surface. The difference in resistivity was demonstrated by the adhesive force on oxide semiconductor films with UV/O_3_ treatment [11,28,29,30]. The parameters are summarized in Table 1. Hence, the static induction transistors with UV/O_3_ treatment led to a higher on-current, larger leakage saturation current, and an Ion/Ioff ratio because of the improved quality of the Ga_2_O_3_ films.

**Figure 5:**
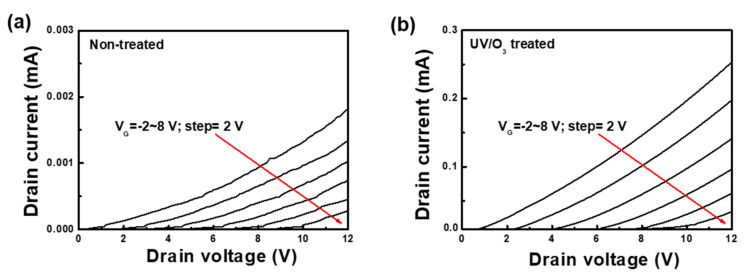
Measured electrical characteristics of Ga_2_O_3_/4H-SiC static induction transistor: I_D_-V_D_ of (**a**) non-treated and (**b**) UV/O_3_ treated for 30 min.

Device characterizations were performed and analyzed on the fabricated SIT devices, which are lateral structures with a vertical back-gate action structure device with a short channel. This gives the SIT a very low resistance. Owing to the low resistance, current saturation is not seen for the case of static I-V measurements [31]. Figure 5 shows the output curve of the I_D_-V_D_ of the Ga_2_O_3_/4H-SiC SITs with the gate voltage varying from −2 to 8 V at 2 V of voltage step. The drain current flowed at zero gate bias (V_G_ = 0 V) and could be increased by applying negative bias or pinch-off through positive bias. The UV/O_3_ treatment reduced the number of oxygen vacancies. Therefore, the number of oxygen vacancy-related charge carrier traps was reduced. This resulted in an increase of the pinch-off and gate currents [32]. The drain current at V_D_ = 12 V was approximately 1.64 × 10^2^ times higher than that after UV/O_3_ treatment. The increase in the output current was considered a result of an increase in the mobility and doping concentration of the Ga_2_O_3_ film due to the reduction in oxygen-related traps. This demonstrated that high-quality Ga_2_O_3_ films were fabricated, and there were significant advantages for transistor operation in terms of output characteristics and on/off ratio.

**Figure 6:**
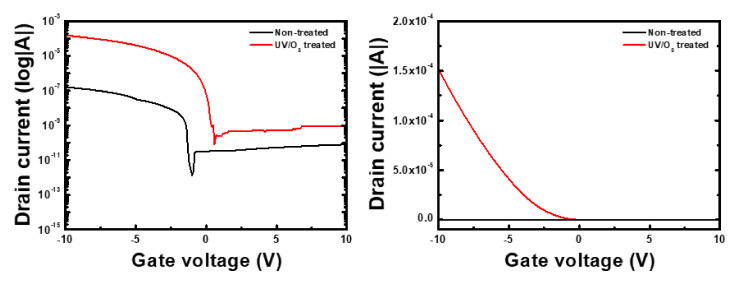
Measured I_D_-V_G_ curves on log scale of Ga_2_O_3_/4H-SiC static induction transistor at V_DS_ = 1 V.

The I_D/G_-V_G_ transfer curves of the Ga_2_O_3_/4H-SiC SITs at V_DS_ = 1 V are shown in Figure 6. Without treatment, the off and on currents were 5.31 × 10^−10^ and 5.26 × 10^−6^ A, respectively, and the Ion/Ioff ratio was 9.92 × 10^3^. However, after UV/O_3_ treatment, the off current was 7.40 × 10^−10^ A while the on-current (Vg = −5 V) was 3.20 × 10^−4^ A, resulting in an Ion/Ioff ratio of 4.32 × 10^5^. The leakage current increased slightly after UV/O_3_ treatment because of the increasing mobility and carrier concentration, but the Ion/Ioff and on-state current improved. The Ga_2_O_3_/4H-SiC SITs showed gate leakage currents (I_GS_) as low as 1.0 × 10^−11^ A at a high gate voltage (10 V), and the depletion layer between n-Ga_2_O_3_ and the p-epi SiC layer at the gate region was a PN junction.

**Figure 7:**
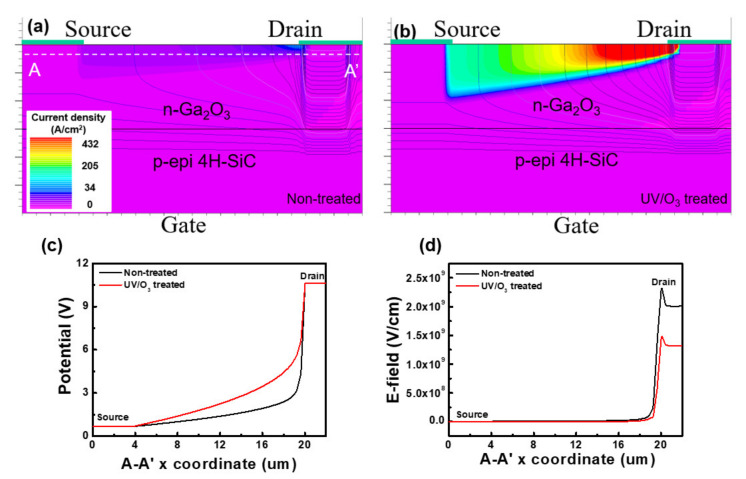
Simulated current density diagram of Ga_2_O_3_/4H-SiC SIT: (**a**) non-treated and (**b**) UV/O_3_-treated. The cutline profile along A-A′: (**c**) potential and (**d**) electric field.

The structure of the SITs was simulated by the 2D-Atlas simulation program on SILVACO to demonstrate the influence of UV/O_3_ treatment. The measured mobility data and doping concentration of Ga_2_O_3_ were used in our simulation. Figure 7a,b shows the current density of the SIT structure at Vs = 0 V, V_D_ = 10 V, Vg = 0 V to compare the device performance with improved parameters through UV/O_3_ treatment. As shown in Figure 7c, the potential slope of the UV/O_3_ treatment device is larger than that of the non-treatment device. Through this data, the concentration of Ga_2_O_3_ in the UV/O_3_ treatment device was higher than that of the non-treatment device. As a result, the area of the E-field was wider. The drain current density increased because the mobility and doping concentration improved. As shown in the structures, the drain current of the UV/O_3_-treated device was approximately 1.34 × 10^2^ times higher than the non-treated device at the same drain voltage. Figure 7c,d shows the potential and electric field distribution at cutline from A to A′.

The slope of the potential at the edge of the drain of UV/O_3_-treated devices was lower than that of non-treated devices. Moreover, the electric field decreased from 2.47 × 10^9^ V/cm to 1.44 × 10^9^ V/cm. Note that the resistivity of Ga_2_O_3_ was decreased by increasing the mobility and doping concentration of n-Ga_2_O_3_.

**Figure 8:**
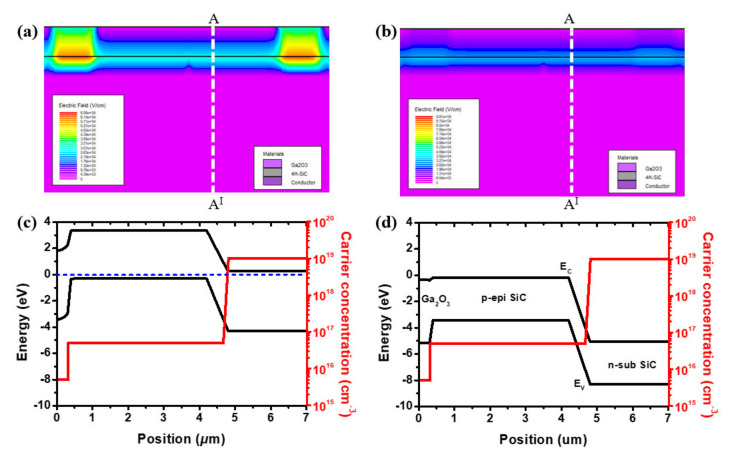
The device structures and energy band diagram of n-Ga_2_O_3_/p-epi/n-sub SiC static induction devices: (**a**) zero gate voltage bias electric field structure, (**b**) applied gate voltage electric field structure, (**c**) zero gate voltage bias energy band, (**d**) applied gate voltage energy band.

The p-epi layer with a highly doped n-type 4H-SiC substrate was used for fabricating the SITs. Using only a p-type SiC substrate, there was one barrier between the n-Ga_2_O_3_ and p-epi layer. The depletion layer was extended at the reverse gate voltage. Conversely, applying a forward bias to the gate shrank the depletion layer and increased the gate leakage current. By using a p-epi layer with a highly n-type doped substrate, the gate leakage current was reduced by an additional barrier between the p-epi layer and highly n-doped substrate. The structure of SITs and energy band diagram of the n-Ga_2_O_3_/p- layer/n+ SiC substrate at zero gate voltage is shown in Figure 8a,c. In Figure 8b,d, when the gate voltage bias was applied, the expanded additional barrier between the p-layer and n+ substrate 4H-SiC restrained the gate current.

## 4. Conclusions

In summary, Ga_2_O_3_/4H-SiC static induction transistors were fabricated and analyzed. The Ga_2_O_3_ films on SiC obtained by UV/O_3_ treatment showed a lower surface roughness and higher mobility and carrier concentration. Filling the radical oxygen atoms into oxygen vacancies at the surface led to an increase in the drain current and on/off ratio at the same gate and drain voltage, and therefore, improved properties in the Ga_2_O_3_ channel. The UV/O_3_-treated devices showed a 1.64 × 10^2^ times higher drain current and an on/off ratio of 4.32 × 10^5^. Furthermore, the devices showed a low gate leakage current of 1.0 × 10^−11^ A at V_GS_ = 10 V by the heterojunction between p-SiC and n-Ga_2_O_3_, which was a clear improvement, compared to the conventional Schottky gate SITs in wide-bandgap oxide semiconductor materials.

## Figures and Tables

**Table 1:** Surface roughness, resistivity, mobility, and carrier concentration parameters obtained from Hall measurement and AFM.

Sample	Surface Roughness (nm)	Resistivity (Ω·cm)	Mobility (cm^2^V^−1^s^−1^)	Carrier Concentration (cm^−3^)
Non-treated	5.213	740.25	2.58	3.37 × 10^15^
15 min treated	4.243	370.76	3.45	4.89 × 10^15^
30 min treated	3.515	319.85	4.01	4.83 × 10^15^

## Data Availability

Data is contained within the article.
